# From PCOS to PMOS: reframing the clinical pathway around insulin sensitivity and weight-neutral metabolic risk

**DOI:** 10.3389/fendo.2026.1909495

**Published:** 2026-09-25

**Authors:** Haoyang Jiao, Hua Guo, Jianfeng Liu

**Affiliations:** 1 China Institute for History of Medicine and Medical Literature, China Academy of Chinese Medical Sciences, Beijing, China; 2 Graduate School, China Academy of Chinese Medical Sciences, Beijing, China

**Keywords:** cardiometabolic risk, clinical pathway, insulin resistance, metabolic risk, polycystic ovary syndrome, polyendocrine metabolic ovarian syndrome, weight-neutral care

## Abstract

Polycystic ovary syndrome (PCOS) is a heterogeneous condition associated with reproductive, metabolic, pregnancy, and psychological morbidity across the life course. Polyendocrine metabolic ovarian syndrome (PMOS) was recently proposed through a multistep global consensus process, with implementation across guidelines and health systems still under way. This Perspective argues that the clinical value of the naming proposal will depend on whether it redirects care beyond ovarian morphology and body size. Insulin resistance can serve as an actionable management hub linking reproductive endocrine dysfunction with longer-term metabolic risk in a substantial subgroup, but it is neither universal, the sole cause of the syndrome, nor a diagnostic criterion. Routine fasting insulin measurement or homeostatic model assessment of insulin resistance should therefore not be introduced as diagnostic requirements. Instead, we propose a weight-neutral, insulin-sensitivity-centered, life-course pathway aligned with existing guideline recommendations. Glycemic status, lipids, blood pressure, cardiovascular risk, pregnancy planning, psychological health, and risk-directed liver assessment should be considered according to clinical guidance without using BMI as an exclusion criterion. Lifestyle intervention should be evaluated through metabolic, functional, reproductive, and quality-of-life outcomes, not weight change alone. Metformin and GLP-1 receptor agonists should remain confined to guideline-supported indications and evidence limitations. Whether PMOS improves risk recognition, guideline adherence, patient experience, or continuity of care remains to be established.

## Introduction: from disease burden to clinical pathway reframing

Polycystic ovary syndrome (PCOS) affects people across the reproductive life course and has reproductive, endocrine, metabolic, and psychological manifestations. The 2023 International Evidence-based Guideline reports a prevalence of approximately 10%–13%, whereas a 2024 meta-analysis estimated a pooled global prevalence of 9.2%, with variation by diagnostic criteria (1, 2). In China, a nationally representative survey estimated a weighted prevalence of 7.8% among women aged 20–49 years in 2020, an increase of nearly 65% over the preceding decade (3). These estimates support viewing PCOS as a common multisystem condition rather than a narrowly ovarian disorder.

The diagnosis also structures clinical attention. The term “polycystic ovary” can suggest a localized cystic disorder, even though ovarian cysts are not the defining pathology and metabolic abnormalities are clinically important. Body size may also influence perceived metabolic risk. Real-world evidence indicates that recorded overweight or obesity is associated with a higher likelihood of diabetes screening in PCOS, suggesting that body-size cues may influence screening behavior (4). However, this evidence does not establish a lean-specific or OGTT-specific practice gap. Patient-centered studies additionally describe delayed diagnosis, inadequate information, generalized lifestyle advice, weight stigma, and fragmented reproductive, metabolic, and psychological care (5, 6).

The 2023 International Guideline already provides the basis for a systemic pathway. Glycemic status should be assessed at diagnosis in all adults and adolescents with PCOS; a 75-g oral glucose tolerance test (OGTT) is the most accurate assessment regardless of BMI; and lipid assessment, blood pressure, cardiovascular risk, pregnancy risk, psychological health, and quality of life are integral to management (1). PMOS was recently proposed through a multistep global consensus process (7). This Perspective does not propose new diagnostic criteria. Rather, it uses the naming debate as an opportunity to examine how guideline-supported care could be reorganized around metabolic risk, reproductive goals, and life-course follow-up.

## PMOS is more than a name change

The potential value of PMOS is not semantic novelty but a change in clinical framing. The Rotterdam-based diagnostic framework identifies PCOS through hyperandrogenism, ovulatory dysfunction, and polycystic ovarian morphology or, in adults, anti-Müllerian hormone as an alternative marker; metabolic abnormalities are not required for diagnosis (1). These criteria should not be altered by inserting insulin resistance as a diagnostic requirement. Nevertheless, a clinical encounter organized only around cycle symptoms, androgenic features, fertility, or ovarian imaging may leave guideline-recommended metabolic and psychological assessment insufficiently integrated.

The 2026 Lancet Health Policy paper proposed PMOS following a multistep process involving 56 academic, clinical, and patient organizations, 14,360 survey responses from affected people and multidisciplinary health professionals, modified Delphi methods, nominal-group workshops, and implementation analyses (7). This constitutes a multi-stakeholder consensus process. It is not equivalent to a clinical-practice guideline, a formal statement from every participating society, completed ICD recoding, or universal use in practice. The process also had limitations, including voluntary non-probability participation and unequal regional and disciplinary representation. Earlier commentaries had already questioned whether the PCOS label accurately represented the condition (8, 9), while patient and professional surveys documented support for reconsidering the name and the features it should convey (5).

Following publication of the consensus report, the Endocrine Society described PMOS as the new name for the condition, ASRM endorsed its use, and ESHRE formally welcomed the terminology and the proposed transition period (10–12). These responses demonstrate early organizational support, but they should not be interpreted as completed incorporation into every clinical guideline, coding system, electronic health record, or national health system. The 2023 International Guideline was published under PCOS and did not create PMOS diagnostic criteria. A three-year transition has been proposed, with exclusive use of PMOS planned for the 2028 International Guideline update; implementation in disease classification and health systems remains ongoing (7, 10).

## Insulin resistance as an actionable management hub, not a single-cause explanation

Insulin resistance is clinically relevant because it can connect reproductive endocrine abnormalities with metabolic risk in a substantial subgroup. Compensatory hyperinsulinemia may increase ovarian theca-cell androgen production, reduce hepatic sex hormone-binding globulin synthesis, and amplify luteinizing-hormone-related steroidogenesis, thereby increasing free androgen exposure and contributing to follicular dysfunction (13–16). PCOS-related insulin signaling is heterogeneous: impaired metabolic signaling can coexist with preserved steroidogenic responses. These mechanisms are consistent with, but do not prove, a single causal pathway for all phenotypes.

Euglycemic-hyperinsulinemic clamp studies support an intrinsic component of insulin resistance that can occur independently of obesity (17, 18). In a large referral cohort, Tosi et al. reported clamp-defined insulin resistance in 74.9% overall, including 59.3% of normal-weight, 77.5% of overweight, and 93.9% of obese participants (19). These estimates are method-, population-, and setting-dependent and should not be treated as fixed prevalence values.

Positioning insulin resistance as an actionable management hub does not imply that it is universal, that it explains all phenotypic heterogeneity, or that it should define the diagnosis. Clinically available insulin assays have limited accuracy and clinical relevance, and the 2023 guideline does not recommend them for routine care (1). Routine fasting insulin or homeostatic model assessment of insulin resistance (HOMA-IR) should therefore not be added to diagnostic criteria. The relevant distinction is between a management framework that attends to insulin sensitivity and an inadequately standardized diagnostic threshold (**Figure 1**).

**Figure 1:**
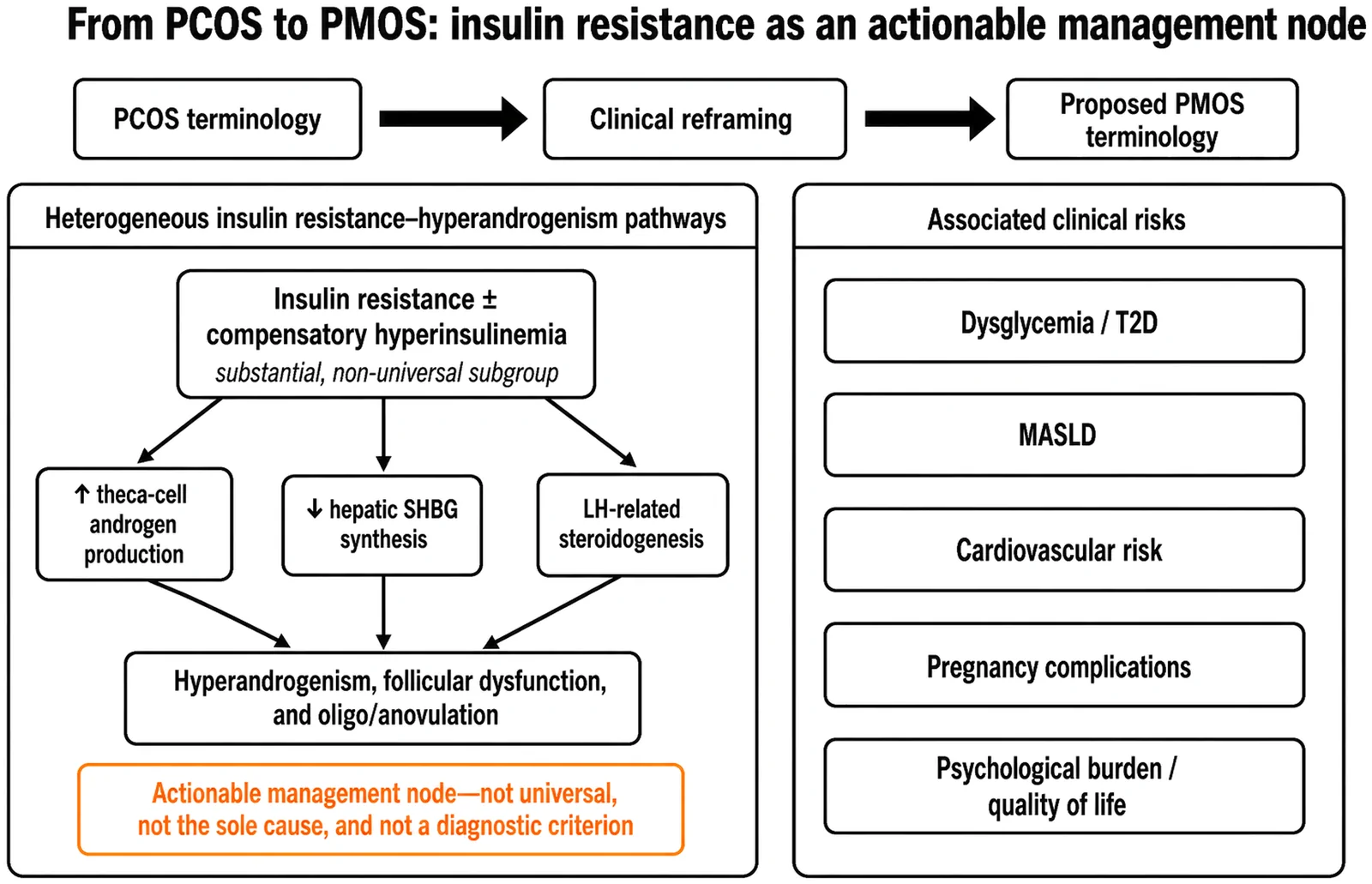
From PCOS to PMOS: insulin resistance as an actionable management node within heterogeneous mechanisms. PMOS was recently proposed through a multistep global consensus process. The figure presents clinical reframing without implying completed adoption. Insulin resistance and compensatory hyperinsulinemia occur in a substantial but non-universal subgroup and are shown as an actionable management node, not a sole cause or diagnostic criterion. Mechanisms and associated risks are heterogeneous. LH, luteinizing hormone; MASLD, metabolic dysfunction-associated steatotic liver disease; PCOS, polycystic ovary syndrome; PMOS, polyendocrine metabolic ovarian syndrome; SHBG, sex hormone-binding globulin; T2D, type 2 diabetes.

## Metabolic risk is not restricted to obesity

Normal-weight or lean phenotypes are not synonymous with absence of metabolic risk. Some normal-weight patients with PCOS have insulin resistance, compensatory hyperinsulinemia, dysglycemia, or altered beta-cell function (19–21). A systematic review and meta-regression found that BMI and ethnicity modify the prevalence of impaired glucose tolerance and type 2 diabetes in PCOS, but obesity did not fully account for risk (22). A Korean national cohort also reported increased type 2 diabetes risk among nonobese women with PCOS compared with matched controls (23). “Normal-weight” and “lean” should not be treated as interchangeable research categories, and neither category defines a uniformly high- or low-risk metabolic phenotype.

Associations with liver, pregnancy, and cardiovascular outcomes also cannot be reduced to BMI alone. Metabolic dysfunction-associated steatotic liver disease (MASLD) is more common in PCOS, with insulin resistance and hyperandrogenism among the associated factors (24, 25). A 2024 meta-analysis reported higher risks of miscarriage, gestational diabetes, hypertensive disorders of pregnancy, preeclampsia, and cesarean delivery; several associations persisted in age- and BMI-matched analyses (26). Cardiovascular evidence supports assessment of risk factors in all patients, while acknowledging that absolute event risk in many premenopausal patients remains low (27).

Notably, a US commercial-claims study found that women with PCOS and recorded overweight or obesity were more likely to undergo diabetes screening than those without such diagnostic codes (79.84%; adjusted OR 1.66, 95% CI 1.62–1.70) (4). Because overweight and obesity were identified from claims-based diagnostic codes rather than measured BMI, and because the analysis was not specific to OGTT use, this finding is suggestive of body-size-influenced screening but does not establish under-screening in normal-weight or lean phenotypes. Together with physician surveys and medical-record studies, it documents an overall guideline–practice gap and infrequent OGTT use, not a measured-BMI disparity (4, 28, 29).

Clinical assessment should therefore follow guideline recommendations rather than use BMI as an exclusion criterion. Glycemic status, lipids, blood pressure, and overall cardiovascular risk should be assessed at diagnosis. A 75-g OGTT is the most accurate glycemic test; fasting plasma glucose and/or glycated hemoglobin may be considered when OGTT cannot be performed, with recognition of reduced accuracy (1). The 2023 PCOS guideline does not recommend universal liver imaging. Liver enzymes, imaging, or fibrosis assessment should instead be guided by symptoms, abnormal liver tests, metabolic risk factors, and applicable liver-disease guidance.

## Weight loss is not synonymous with insulin sensitization

Weight reduction can improve insulin sensitivity and other outcomes for some higher-weight patients, but weight loss and insulin sensitization are not interchangeable endpoints. When success is defined only by weight change, clinically meaningful changes in fitness, sleep, diet quality, body composition, menstrual function, metabolic markers, or quality of life can be overlooked. A weight-neutral approach does not prohibit weight management; it means that access to risk assessment and appropriate care is not conditional on body size or weight loss.

The 2023 guideline recommends lifestyle intervention for all patients to improve metabolic health, central adiposity, lipid profile, quality of life, and weight management when relevant (1). Systematic reviews support benefits for cardiorespiratory fitness, body composition, and some insulin-resistance-related outcomes, without establishing one universally superior exercise modality (30, 31). Trials have reported reproductive or metabolic benefits in both overweight and non-overweight participants (32, 33). Conversely, supervised exercise in lean hyperandrogenic women improved maximal oxygen uptake without improving whole-body insulin action in one study (34). This finding illustrates heterogeneity of response, not futility of exercise, and supports monitoring outcomes aligned with the individual’s goals.

Metformin should be positioned as a metabolic therapy rather than a weight-loss drug (15, 35). The guideline states that metformin should be considered in adults with PCOS and BMI ≥25 kg/m² for anthropometric and metabolic outcomes and may be considered when BMI is <25 kg/m², while acknowledging limited evidence (1). Its use may be particularly relevant when impaired glucose tolerance or additional diabetes risk is present. Decisions should incorporate gastrointestinal adverse effects, vitamin B12 risk, contraindications, patient preferences, and the fact that metformin should not displace more effective ovulation-induction therapy when infertility is the principal goal. Evidence comparing metformin with combined oral contraceptive pills remains heterogeneous and goal-dependent (36).

GLP-1 RAs require narrower positioning. The guideline allows consideration of anti-obesity medications, including liraglutide and semaglutide, for higher-weight adults with PCOS in accordance with general population guidance and alongside active lifestyle intervention (1). Where pregnancy is possible, effective contraception is required because pregnancy safety data are insufficient. Gradual dose escalation, gastrointestinal adverse effects, the potential need for long-term treatment, and weight regain after discontinuation should be discussed. Systematic reviews support weight and waist-circumference reduction in studied higher-weight populations, but PCOS-specific cardiovascular outcomes, normal-weight or lean phenotypes, and weight-loss-independent benefits remain uncertain (37–39). GLP-1 RAs are therefore not foundational therapy for all patients with PCOS or PMOS.

## A weight-neutral, insulin-sensitivity-centered clinical pathway

The proposed pathway is metabolically alert without labeling every patient as insulin resistant. It is an organization of guideline-supported care, not a new diagnostic standard. Entry begins with an established PCOS diagnosis or a suspected phenotype requiring diagnostic evaluation; neither BMI nor ovarian ultrasonography alone determines metabolic assessment.

Baseline assessment for all should include glycemic status, preferably by 75-g OGTT; blood pressure; lipid profile; body size and waist circumference as descriptive and treatment-relevant measures rather than exclusion criteria; family history; prior pregnancy complications; pregnancy intentions; symptoms of obstructive sleep apnea; depression and anxiety screening; quality-of-life priorities; medication preferences; and contraceptive needs (1). Liver assessment is risk directed rather than universal. For patients planning pregnancy or fertility treatment, OGTT should be offered before conception or treatment; if not completed, it should be offered at the first antenatal visit and repeated at 24–28 weeks (1).

Intervention domains should be selected according to metabolic findings, the patient’s primary clinical goal, life stage, body-size phenotype, comorbidities, and preferences; they are not a fixed sequence. Lifestyle intervention can target diet quality, aerobic and resistance activity, sleep, fitness, and behavioral support. Combined oral contraceptive pills and, where indicated, anti-androgen therapy can address cycle regulation and hyperandrogenic symptoms. Metformin can be considered according to metabolic indications and the evidence limitations described above. Letrozole remains first-line pharmacologic ovulation induction for anovulatory infertility when no other infertility factors are present. GLP-1 RAs are reserved for selected higher-weight adults under anti-obesity treatment guidance and are not indicated solely because a patient has PCOS or PMOS.

Follow-up should be longitudinal and risk based. Glycemic status should be reassessed every 1–3 years according to individual diabetes risk; lipid and blood-pressure follow-up should reflect baseline findings and overall cardiovascular risk (1). Depression and anxiety screening should be integrated into care because both are more prevalent in PCOS (40). Patients should be informed of the increased risk of endometrial hyperplasia and cancer while being reassured that absolute risk remains low and routine screening is not recommended. Long-standing untreated amenorrhea, higher weight, type 2 diabetes, and persistent endometrial thickening warrant additional vigilance and preventive management (1, 41). **Figure 2** summarizes the proposed pathway.

**Figure 2:**
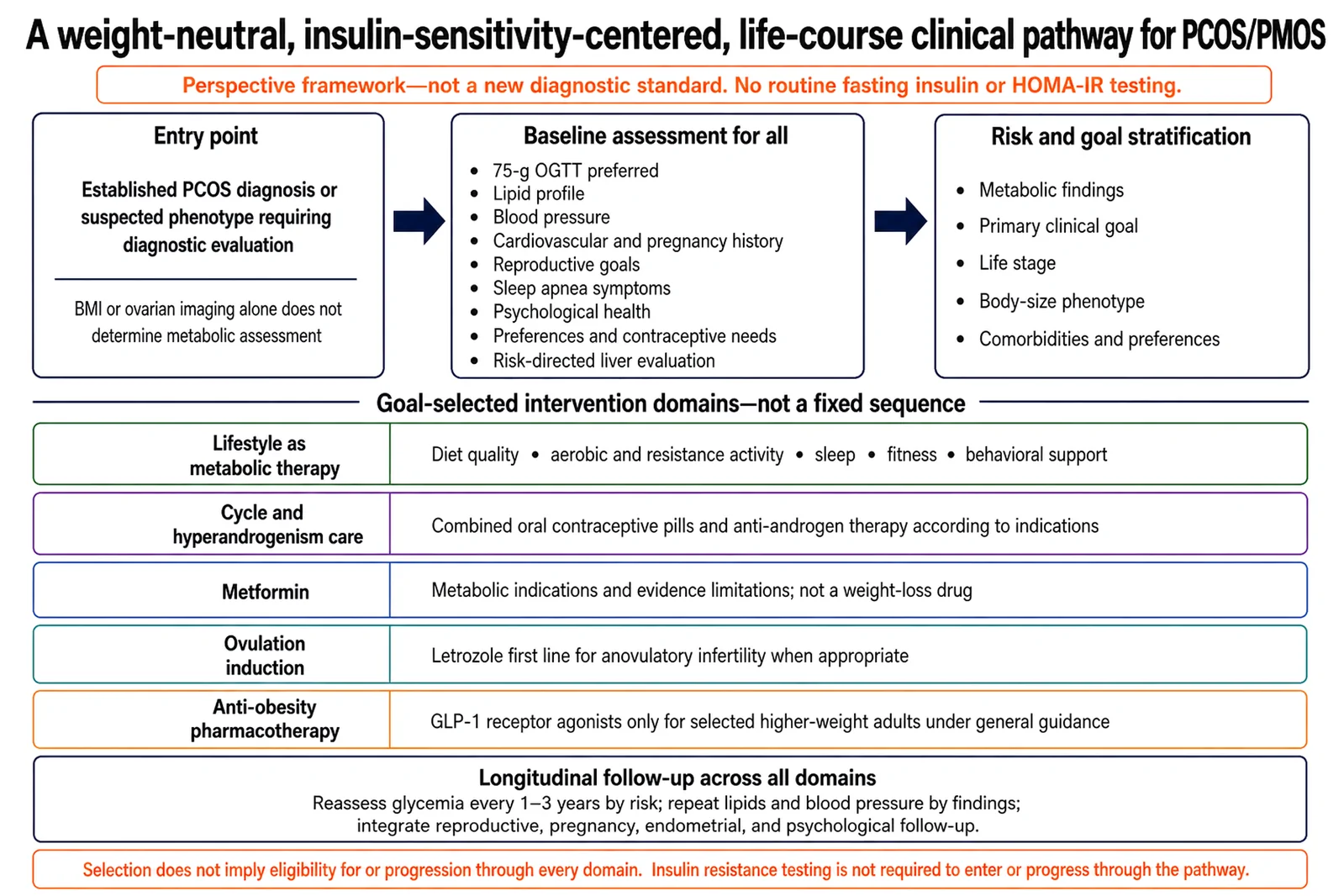
A weight-neutral, insulin-sensitivity-centered, life-course clinical pathway for PCOS/PMOS. This Perspective framework reorganizes guideline-supported care and is not a new diagnostic standard. Baseline assessment is offered across body-size phenotypes; liver evaluation is risk directed. Interventions are goal-selected domains rather than a fixed sequence, and longitudinal follow-up applies across all domains. GLP-1 receptor agonists are limited to selected higher-weight adults under general anti-obesity guidance. Routine fasting insulin or HOMA-IR testing is not required.

## Scope and limitations of this perspective

This Perspective is a conceptual and clinically oriented synthesis, not a systematic review, clinical-practice guideline, or proposal for new diagnostic criteria. The supporting literature was selected to examine specific claims about nomenclature, metabolic risk, screening implementation, and treatment positioning; therefore, absence of a study from this discussion should not be interpreted as evidence of absence. Real-world studies document incomplete metabolic screening and low OGTT use in PCOS overall, but no study directly comparing OGTT use across measured BMI categories was identified. Evidence for insulin-resistance mechanisms and treatment response is heterogeneous across methods, populations, and phenotypes, and the proposed pathway has not been prospectively evaluated. The effects of PMOS terminology on guideline adherence, coding, stigma, patient understanding, resource use, and clinical outcomes also remain to be established.

## Research agenda: from nomenclature to testable implementation

Mechanistic and clinical research should improve insulin-resistance phenotyping without prematurely converting research assays into diagnostic tests. Priorities include validation of clinically meaningful metabolic subtypes; characterization of normal-weight and lean phenotypes, including insulin secretion and clearance; and trials that distinguish weight-loss-dependent from weight-loss-independent effects of exercise, metformin, GLP-1 RAs, and combination strategies. Long-term, phenotype-stratified studies are needed for cardiovascular events, liver outcomes, pregnancy outcomes, and offspring health.

Implementation questions are equally important. Audits, registries, claims analyses, and electronic health record studies should report OGTT and broader metabolic-screening rates across measured BMI and body-composition categories, while avoiding the assumption that normal-weight and lean phenotypes are equivalent. Clinician surveys and vignette experiments should test whether body size changes screening decisions after other risk factors are held constant. These studies should also identify barriers related to specialty ownership, appointment time, test burden, reimbursement, access, and patient preference.

The PMOS transition itself should be evaluated rather than assumed to be beneficial. Studies should determine whether the terminology improves guideline-concordant assessment, continuity between reproductive and metabolic care, and patient understanding; whether it reduces stigma or creates new forms of metabolic labeling and overmedicalization; and how it affects coding, insurance coverage, electronic records, clinical decision support, and research indexing. Predefined implementation outcomes could include the proportion receiving baseline and interval glycemic assessment, use of OGTT when indicated, lipid and blood-pressure assessment, preconception testing, psychological screening, risk-directed liver evaluation, documented shared decision-making, referral completion, patient-reported understanding, stigma measures, and loss to follow-up.

These questions distinguish three levels of claim: established metabolic risk across body-size phenotypes; guideline-based inference that BMI should not exclude assessment; and the authors’ proposal that PMOS could serve as an implementation prompt. Whether the proposed pathway or terminology improves outcomes remains to be established.

## Conclusion

The PMOS naming initiative creates an opportunity to reconsider how PCOS care is organized, but a new term alone does not change clinical outcomes. Insulin resistance can be an actionable management hub in a substantial subgroup without becoming a universal cause or diagnostic criterion. A weight-neutral, insulin-sensitivity-centered pathway is therefore best understood as an implementation of existing evidence: metabolic and reproductive assessment should be integrated, body size should not exclude guideline-recommended testing, treatment should follow clinical goals and indications, and follow-up should extend across the life course. The proposed pathway requires prospective evaluation. Its success should be judged by measurable improvements in risk recognition, reproductive and metabolic care, patient experience, and continuity—not by adoption of the acronym alone.

## Data Availability

The original contributions presented in the study are included in the article/supplementary material. Further inquiries can be directed to the corresponding authors.
